# Health literacy predicts participant understanding of orally-presented informed consent information

**DOI:** 10.15761/CRT.1000105

**Published:** 2015-04-19

**Authors:** Raymond L Ownby, Amarilis Acevedo, Kenneth Goodman, Joshua Caballero, Drenna Waldrop-Valverde

**Affiliations:** 1 Department of Psychiatry and Behavioral Medicine, Nova Southeastern University, USA; 2 Center for Psychological Studies, Nova Southeastern University, USA; 3 Bioethics Programs, University of Miami School of Medicine, US; 4 College of Pharmacy, Department of Pharmacy Practice, Nova Southeastern University, USA; 5 Nell Hodgson Woodruff School of Nursing, Emory University, USA

## Abstract

Informed consent for participation in studies with human subjects is a critically important aspect of clinical research, but research has shown that many potential subjects do not understand information relevant to their participation. A better understanding of factors related to participant understanding of study-related information is thus important. As part of a study to develop a new measure of health literacy, participants viewed a 50 second video in their preferred language (Spanish or English) of a clinician presenting informed consent information. They then responded to six questions about it. In progressively more complicated regression models, we evaluated the relation of demographic variables, general cognitive ability, and health literacy to participants’ recall of the information. In a model that only included demographic variables, Spanish language, black race and older age were associated with poorer performance. In a model that included the effects of general cognitive ability and health literacy as well as demographics, education and health literacy were related to performance. Informed consent interventions that take potential research subjects’ levels of health literacy into account may result in better understanding of research-related information that can inform their decision to participate.

## Introduction

Giving potential research subjects the information they need to make informed choices about participation in clinical studies is a key part of the informed consent process with crucial ethical and practical implications for the conduct of clinical research. The informed consent process is essential in ensuring that potential participants have the information they need to make decisions about becoming involved in a clinical study, and helps investigators to meet their ethical obligation to respect participants’ autonomy while not harming them [1,2]. In spite of this importance, however, investigations show that participants often have a limited understanding of study information after it is presented during the informed consent process [3–7]. Findings among research subjects are mirrored by findings on patients’ understanding of informed consent information related to medical treatment [8–10] and on information provided by clinicians during regular clinical encounters [11]. These results suggest that understanding what affects participants’ understanding of participation and how they use this understanding to make decisions may be important in improving the informed consent process in clinical research.

Health literacy is defined as the ability to obtain and use health information to make decisions [12]. In recent years, it has increasingly been recognized as an important factor in patient health. It has been related to patient’s health status, self-care behaviors, and even outcomes such as risk for mortality. Lower levels of health literacy have been related to health service utilization, lower adherence to treatment recommendations, and poorer health-related quality of life [13–15]. They have even been related to increased risk of mortality [16–18]

Health literacy is an essential skill for persons considering participation in a clinical research study [19]. Understanding an informed consent form requires health literacy skills, yet many standard forms are too difficult for participants to understand [20,21]. Williams et al. showed that 60% of a group of patients at two large hospitals serving minority and low-income patients did not understand a standard informed consent form [22]. Observations such as these have led several authors to argue for the importance of health literacy in the consent process [19,23,24]. Previous research has shown that the same groups known to have lower levels of health literacy [25] may also have difficulty in understanding information related to informed consent [26,27]. Members of racial or ethnic minorities and the elderly have limited health literacy skills [25] that may be related to their ability to understand information presented during the informed consent process. Researchers have shown that being older and having fewer years of education are associated with poorer understanding of informed consent materials [27,28].

Research on the extent to which language, minority status, education and age are related to understanding in the consent process is limited. In a review of 54 studies of interventions to improve the informed consent process in research, Nishimura *et al.* [6] note that literacy level or income may have been a factor in the heterogeneity they found in study results but found few studies that reported on these factors. They conclude that findings on interventions may not apply to persons with low literacy or who are socially disadvantaged, and that additional research on these factors is needed. Health literacy is likely to be related to understanding informed consent yet empirical evidence for this association is limited. The purpose of this study was to evaluate the relation of health literacy to understanding orally-presented informed consent information.

## Method

### Overview

Participants were drawn from a larger study whose purpose was to develop a computer-delivered measure of health literacy, described in greater detail elsewhere [29]. As part of materials developed for this project, a brief video was designed to simulate an informed consent encounter. The video consisted of a female investigator explaining a portion of the information usually provided in review of study characteristics, such as the reason for the possible participant being asked to be in the study, its risks, and alternative treatments. Participants also completed cognitive assessments and FLIGHT/VIDAS, the new measure of health literacy [29]. Factors that predicted participants’ ability to remember information from the video were assessed in multivariable regression models.

### Informed consent process

Potential participants were first screened by telephone; their verbal consent for completing screening procedures was first obtained. The screening process included administration of two brief cognitive measures assessing concentration, orientation, and memory in order to help ensure that potential participants did not have significant cognitive deficits that might preclude their participation. Participants who met screening criteria were scheduled for an in-person study visit. Prior to completion of any study procedures for US-born English-speaking participants, written informed consent was obtained. For Spanish-speakers who indicated they spoke both Spanish and English, verbal consent was obtained for additional screening of language proficiency. This was accomplished through administration of timed reading and listening comprehension measures in both languages; the language in which the potential participant was most proficient was that used for the informed consent process and for further study participation. Prior to study participation, researchers reviewed study information, provided the participant sufficient time to read the written informed consent form, and answered participant questions. No participant declined to sign the written informed consent form. All study procedures were conducted under a protocol approved by the Institutional Review Board of Nova Southeastern University.

### Procedures

Participants completed two testing sessions. One consisted of individually-administered measures of cognitive ability and academic skills while the other included computer-based administration of measures of mood, health symptoms and conditions, health-related quality of life, and ratings of the usability of the health literacy measure. This session also included computer administration of the 98 questions in the new health literacy measure. Participants were allowed to complete both sessions in a single day, but were encouraged to take breaks as needed during assessments and all took a break for lunch that was provided via a voucher for a local restaurant. Order of assessment sessions was randomized to take possible order effects into account.

### Measures

Participant age, education, gender, and race were recorded as was the language (Spanish or English) in which measures were administered. *General cognitive abilities* [30,31] were assessed using the Verbal Composite score of the Woodcock-Johnson Psycho-Educational Battery for English speakers and the Woodcock-Muñoz Psycho-Educational Battery for Spanish speakers (WJPEB/WMPEB; [32,33]). These measures evaluate word knowledge, general information, and verbal reasoning in a series of tasks that yields a single standard score. These measures were used because they were available in both Spanish and English with norms based on both language groups.

As noted above, the development of the FLIGHT/VIDAS health literacy scale has been reported in more detail elsewhere [29] and will be only briefly recounted here. A group of 208 candidate items were pilot tested with groups of Spanish and English speaking volunteers. Participants were interviewed about item content format, and preliminary psychometric analyses of how the items functioned in both languages and of item discrimination and difficulty allowed us to choose a group of 98 items for the second phase of the study. In this next development phase, data from a larger group of Spanish and English-speaking community-dwelling volunteers has allowed further analyses that support the construction of subscales for the measure. These assess general health literacy, numeracy, listening comprehension, and health knowledge. FLIGHT/VIDAS is computer-administered and scored, with multimedia, Internet-related, and video stimulus materials.

*General health literacy* was assessed with a group of 40 test items based on a variety of contents in several different question formats. Items incorporated understanding short passages of text as well as the ability to integrate information from text with data from graphs or tables. *Numeracy* was assessed in a series of items that integrate understanding figures and tables with arithmetic computation and quantitative reasoning. Numeracy scale items also assessed understanding of relative probabilities (such as risk for a side effect) and decision making based on relative probabilities. *Health knowledge* was assessed with another subscale of the new measure designed to be a measure of general health-related information. It comprises 15 questions, all of which assess knowledge of a specific fact, such as a disease risk factor or a specific drug treatment for a disease, and do not require substantial reading or numeracy skills, as for example, “Cholesterol is measured with a …” *Listening*. As part of the new measure, several other questions were developed that required participants to listen to information provided via videos or a slide presentation and respond to questions. Participants performance on these other questions were summed to create a measure of listening comprehension for health-related material not directly related to informed consent.

### Informed consent video

For both Spanish and English versions, the video consisted of a female researcher explaining informed consent information to a potential participant concerning a trial of a fictitious cholesterol-lowering medication. Information included (1) a request for participation in a clinical research study; (2) reason for participation (inadequate control of cholesterol level); (3) participation is voluntary; (4) common side effects; (5) serious side effects; (6) strategies for monitoring liver function via blood tests; (7) alternatives to being in the study, and (7) that if the participant were to choose not to participate, that decision wouldn’t affect his or her current treatment.

All materials were presented on a 20 inch (diagonal) touch screen computer (HP TouchSmart; Palo Alto CA: Hewlett-Packard Corporation). Participants viewed the video once while hearing the voice of the nurse over speakers integrated into the computer. They then proceeded to additional computer screens that presented follow-up questions one at a time. Participants indicated answers by tapping on the chosen response on the computer screen. Six questions assessed information contained in the video. They ranged in difficulty from a question assessing a straightforward fact (reason for the person being asked to participate) to requiring judgment about which of several side effects was most serious (rhabdomyolysis) or the likelihood of a rare but serious side effect (requiring a choice among various probabilities and the interpretation of the word “rare”).

### Analyses

Correct scores for each question were summed to yield a single score for each participant. Linear regression models included predictor variables in blocks to assess (1) demographic variables, including language, age, gender, race, and education; (2) general cognitive ability; (3) health literacy. Blocks of variables were included in regression models in progressively more complex models. Change in the amount of variability in informed consent material understanding accounted for was evaluated via the R2 value associated with each model. All analyses were completed using SPSS version 21 (IBM, Inc., Montauk, NY).

## Results

Descriptive statistics for study participants are presented in Table 1. For this study, we intentionally recruited participants across a wide age range (18 to 85); the average age for the combined sample of Spanish and English speakers was 51.0 years with a standard deviation of 16.7. The sample included individuals whose levels of formal education ranged from 3 to 20 years. The combined sample mean years of education was 12.9 with a standard deviation of 2.6. The English-speaking group included both blacks and whites, while all Spanish-speaking participants described themselves as Hispanic and white. The average total score on the informed consent questions was for the combined sample was 3.7 with a standard deviation of 1.1, and participant scores ranged from 0 to 6 correct. Table 2 presents item statistics for the six informed consent questions by language. Language-based differences emerged on most items favoring those who completed them in English, with the exception of one question (worst risk of being in the study) which was answered correctly significantly more often by Spanish speakers.

Regression models are presented in Table 3. In Model 1, with only demographic variables and education, Spanish language, black race, and greater age were associated with lower scores on the informed consent items, and a more years of education were associated with higher scores. This model accounted for 15% of the variability in performance on the informed consent items. In Model 2, which included general cognitive ability, age and race but not language was associated with lower scores and education with higher scores on the informed consent items. While a statistically significant predictor, the addition of general cognitive ability to the regression only increased the percentage of variability accounted for in the model 2 by 2%. In Model 3, which added measures of health literacy, education, health literacy and numeracy were associated with higher scores. Race, language, and age were no longer associated with recall of informed consent information in this model that now accounted for 29% of variability in scores.

## Discussion

The purpose of these analyses was to empirically evaluate whether participants’ health literacy was related to their memory for orally-presented informed consent information. Results show that most participants were able to recall some specific information relevant to their participation in a clinical study but that their recall was less than perfect. These findings are consistent with those reported by others, who have shown that orally-presented information in informed consent interviews is typically only partially remembered [6]. They are also consistent with studies of patients’ memory for information presented during informed consent procedures prior to medical procedures [9] and in clinical encounters more generally [11].

In the regression model that included only demographic variables and education, participant characteristics associated with lower performance were speaking Spanish, being Black, and being older, while higher levels of education were associated better performance. In a more complex model that took cognition and health literacy into account, we found that their inclusion reduced the effect of race and language on participants’ performance, but that education remained important. This finding suggests that race and ethnicity in themselves may not be as important as education, health literacy, and their interaction in predicting the understanding of informed consent information. Since research on health literacy and outcomes has sometimes only examined the effects of demographic variables without taking cognitive function into account, this finding may have implications for understanding the relations among race and ethnicity and health status and outcomes.

Results of the models also yield insights into factors that may have an impact on language and race-related differences in performance on the informed consent related test items. The model that included only demographic variables, for example, indicated that blacks and Spanish-speakers remembered less of the video content. When health literacy was taken into account, however, the effects of race and language were no longer statistically significant. This finding can be interpreted as suggesting that while race and language appear directly related to performances, the observed effects may actually arise from health literacy and the interaction of health literacy with education. This finding has practical implications, since a number of studies have shown that interventions to improve health literacy can be effective [34]. This raises the possibility that race- and ethnicity-related differences in understanding during the informed consent process might be addressed by interventions to improve their health literacy.

An alternative strategy might be to tailor the informed consent process to possible participants’ levels of health literacy. In other studies, for example, we have shown that computer-delivered tailored information interventions may be effective in improving patient knowledge and adherence behavior [35,36]. Other authors have argued that computer-delivered interventions may be an effective strategy to address race- and ethnicity-related health disparities [37].

Limitations of this study should be acknowledged. While the brief video simulated the type of encounter a possible subject might have during the informed consent process, it only represented a small part of the longer process during which potential subjects review a written informed consent form, ask questions, and interact more extensively with a researcher. While these results highlight factors related to our participants’ understanding of orally-presented information, they cannot be interpreted as reflecting the entire informed consent process. Taken together with others’ finding that written consent forms are often too difficult for many participants to understand [20,21], however, these results underline the importance of providing possible subjects the opportunity to interact with the investigator to ensure their understanding. This observation is consistent with the finding that extended discussion during the informed consent process was associated with subjects’ better understanding [6]. Another limitation is related to the nature of the sample used in the study. As the purpose of the core study from which these data are drawn is to develop a new measure of health literacy that would be broadly useful in a variety of situations, we recruited a sample from a wide range of backgrounds, education levels, and ages. Especially in our inclusion of participants who are young and healthy this sample does not represent persons who might become involved in clinical research. It must also be recognized that other unmeasured variables not included in these analyses might have been important in understanding the importance of health literacy for understanding informed consent information. Our selection of variables, too, may have biased our findings by focusing on some variables while not measuring others.

These results underscore the importance of recognizing that potential clinical research subjects may be less likely to understand information related to their participation in a study. Although results suggest that education and health literacy may be the most important factors in potential participant understanding, in regular clinical research practice it should still be recognized that Spanish-speaking subjects may not remember information as well as those who speak seak English, even when information is presented in Spanish. Blacks and older persons may also be unable to understand information as well as those who are white and younger. Persons who are most at risk for health-related problems and are potentially vulnerable may thus be at a disadvantage in making informed decisions about their participation. While it may be possible to develop interventions to address the issues identified in this study, researchers should be aware of the difficulties some participants may have in understanding material presented during the informed consent process and be vigilant in ensuring that their rights are adequately protected.

## Figures and Tables

**Table 1:** Description of sample.

	English Mean (SD)	Spanish Mean (SD)
N	150	184
Age	52.9 (17.5)	49.3 (15.8)
Years of Education	13.5 (2.3)	12.4 (2.8)
General Cognition	95.9 (10.5)	89.4 (9.0)
Health Literacy^b^	24.8 (5.5)	21.0 (5.9)
Numeracy^b^	15.6 (5.1)	12.5 (5.2)
Listening^d^	7.3 (1.6)	7.3 (1.6)
Knowledge^e^	8.7 (3.0)	7.5 (2.6)
Informed Consent^f^	3.8 (1.0)	3.6 (1.3)
	English *N*	Spanish *N*
Gender: Men/Women	65/85	73/111
Race: White/Black	83/67	184/0^f^

a General cognitive abilities from Woodcock-Johnson or Woodcock-Muñoz Psycho-Educational Batteries.

b General health literacy score from FLIGHT/VIDAS.

c Health numeracy score from FLIGHT/VIDAS.

d Listening scale representing sum of correct answers to other video items that did not include informed consent related material.

e General health knowledge scale representing sum of correct answers to 15 fact-based health knowledge questions.

f Informed consent score represents the total correct answers to six questions.

**Table 2:** Item description and comparison by language.

	English Mean (SD)	Spanish Mean (SD)	Difference	*t*	*df*	*p*
Why would someone be asked to be in the study?	0.90 (0.30)	0.88 (0.33)	0.02	0.57	332	0.57
How likely is the severe side effect of severe muscle problems?	0.17 (0.38)	0.10 (0.31)	0.07	1.87	332	0.06
What is the worst risk of being in the study?	0.27 (0.45)	0.41 (0.49)	−0.13	−2.58	332	0.01
What does "voluntary" mean?	0.96 (0.20)	0.85 (0.36)	0.11	3.29	332	< 0.001
How will they check for liver problems?	0.89 (0.32)	0.80 (0.40)	0.08	2.05	332	0.04
What happens if someone doesn't want to be in the study?	0.63 (0.48)	0.49 (0.50)	0.14	2.66	332	0.01
Total score	3.83 (0.97)	3.54 (1.25)	0.29	2.31	332	0.02

**Table 3:** Regression models.

	Model 1: Demographics				Model 2: General Cognition				Model 3: Health Literacy			
	*F* (5, 317)=12.61, *p*<0.001; R^2^=0.15				*F* (6, 316)=12.10, *p*<0.001; R^2^=0.17				*F* (11, 311)=11.80, *p*<0.001; R^2^=0.29			
	B	SE	*t*	*p*	B	SE	*t*	*p*	B	SE	*t*	*p*
**(Constant)**	3.56	0.69	5.16	< 0.001	1.62	0.96	1.69	0.09	−0.27	1.33	−0.20	0.84
**Spanish Language**	−0.40	0.15	−2.64	0.01	−0.25	0.16	−1.59	0.11	0.02	0.15	0.12	0.91
**Female Gender**	0.09	0.12	0.77	0.44	0.17	0.12	1.39	0.16	0.17	0.12	1.51	0.13
**Black Race**	−0.53	0.19	−2.76	0.01	−0.40	0.20	−2.03	0.04	−0.09	0.19	−0.50	0.62
**Age**	−0.01	0.00	−2.99	0.003	−0.01	0.00	−3.12	0.002	0.00	0.00	−0.73	0.46
**Education**	0.14	0.02	6.12	< 0.001	0.12	0.02	4.78	< 0.001	0.22	0.08	2.82	0.01
**General Cognition**					0.02	0.01	2.86	0.01	−0.01	0.01	−1.03	0.31
**Health Literacy**									0.11	0.05	2.34	0.02
**Numeracy**									0.08	0.02	4.42	< 0.001
**Listening**									0.02	0.04	0.47	0.64
**Health Knowledge**									0.04	0.03	1.19	0.23
**Health Literacy × Education**									−0.01	0.00	−2.23	0.03
